# Induction of mitochondrial reactive oxygen species production by GSH mediated S-glutathionylation of 2-oxoglutarate dehydrogenase

**DOI:** 10.1016/j.redox.2016.02.002

**Published:** 2016-02-17

**Authors:** Ryan J. Mailloux, D. Craig Ayre, Sherri L. Christian

**Affiliations:** Department of Biochemistry, Memorial University of Newfoundland, St. John's, Newfoundland, Canada

**Keywords:** Ogdh, 2-oxoglutarate dehydrogenase, ROS, reactive oxygen species, Grx2, glutaredoxin-2, H_2_O_2_, hydrogen peroxide, SOD, superoxide dehydrogenase, 2-oxo, 2-oxoglutarate, Krebs cycle, Reactive oxygen species, Redox signaling, Glutathione, 2-Oxoglutarate dehydrogenase, S-glutathionylation, Glutaredoxin-2

## Abstract

2-Oxoglutarate dehydrogenase (Ogdh) is an important mitochondria redox sensor that can undergo S-glutathionylation following an increase in H_2_O_2_ levels. Although S-glutathionylation is required to protect Ogdh from irreversible oxidation while simultaneously modulating its activity it remains unknown if glutathione can also modulate reactive oxygen species (ROS) production by the complex. We report that reduced (GSH) and oxidized (GSSG) glutathione control $O_{2}^{∙-}$/H_2_O_2_ formation by Ogdh through protein S-glutathionylation reactions. GSSG (1 mM) induced a modest decrease in Ogdh activity which was associated with a significant decrease in $O_{2}^{∙-}$/H_2_O_2_ formation. GSH had the opposite effect, amplifying $O_{2}^{∙-}$/H_2_O_2_ formation by Ogdh. Incubation of purified Ogdh in 2.5 mM GSH led to significant increase in $O_{2}^{∙-}$/H_2_O_2_ formation which also lowered NADH production. Inclusion of enzymatically active glutaredoxin-2 (Grx2) in reaction mixtures reversed the GSH-mediated amplification of $O_{2}^{∙-}$/H_2_O_2_ formation. Similarly pre-incubation of permeabilized liver mitochondria from mouse depleted of GSH showed an approximately ~3.5-fold increase in Ogdh-mediated $O_{2}^{∙-}$/H_2_O_2_ production that was matched by a significant decrease in NADH formation which could be reversed by Grx2. Taken together, our results demonstrate GSH and GSSG modulate ROS production by Ogdh through S-glutathionylation of different subunits. This is also the first demonstration that GSH can work in the opposite direction in mitochondria-amplifying ROS formation instead of quenching it. We propose that this regulatory mechanism is required to modulate ROS emission from Ogdh in response to variations in glutathione redox buffering capacity.

## Highlights

• ROS formation by Ogdh is controlled by glutathione.
• GSH amplifies ROS production by Ogdh.
• Ogdh is S-glutathionylated by GSH.
• Grx2 deglutathionylates Ogdh.

## Graphical abstract

**Figure:**
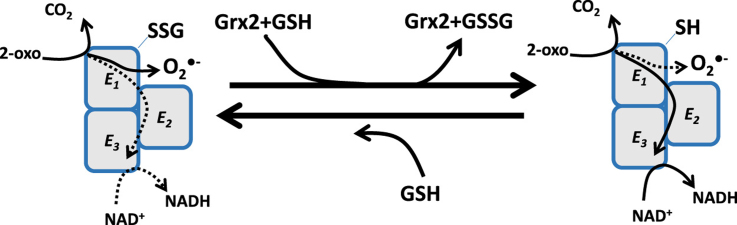

## 1 Introduction

**Fig. 1:**
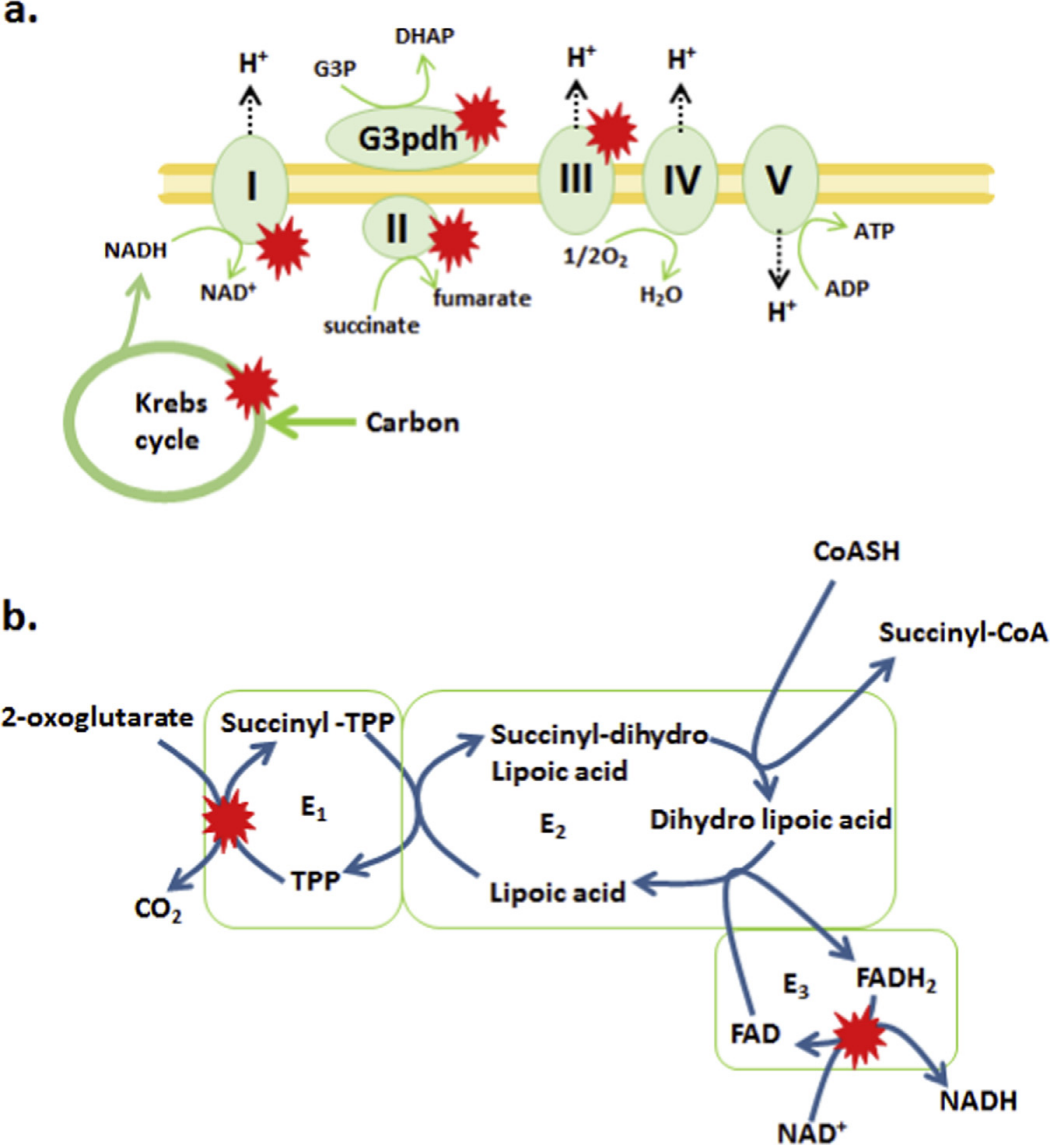
Oxidative metabolism and ROS production by 2-oxoglutarate (Ogdh). (A) Schematic illustration of the oxidation of carbon and oxidative phosphorylation in mitochondria. Electrons liberated from the oxidation of nutrients in the Krebs cycle are ferried through the respiratory chain the terminal electron acceptor di-oxygen (O_2_). Electron movement is coupled to the formation of a membrane potential composed of electrochemical difference in protons which drives ATP production. Also depicted are the sites of ROS formation (red stars) in the nutrient oxidation and respiratory chain. Note that the red star in the Krebs cycle represents ROS formation by Ogdh and Pdh, respectively. Sites for ROS production in the electron transport chain include *sn-*glycero-3-phosphate dehydrogenase (G3pdh), Complexes I, II, and III. For simplicity, other ROS forming sites including electron-transferring flavoprotein-ubiquinone oxidoreductase (Etfqo), proline dehydrogenase (Prodh), dihydrolipoamide dehydrogenase (Dhodh), 2-oxoadipate dehydrogenase (Dhtkd1), and sulfide-quinone oxidoreductase (Sqo), were omitted. The reader is encouraged to consult these articles on the 12 sites of production in mitochondria [3], [20], [34], [35]. (B) Generation of $O_{2}^{∙-}$/H_2_O_2_ by 2-oxoglutarate dehydrogenase Ogdh complex. The complex is composed of E_1_ (2-oxoglutarate decarboxylase), E_2_ (dihydrolipoamide succinyl transferase), and E_3_ (dihydrolipoamide dehydrogenase) which couple 2-oxoglutarate oxidation to NADH formation. The enzyme complex contains multiple copies of each subunit indicated by a green outline. 2-Oxoglutarate is first oxidized by E_1_ resulting in the liberation of CO_2_ and the succinylation of thiamine pyrophosphate (TPP). The succinyl moiety is then transferred to a vicinal thiol on lipoamide in the E_2_ subunit which then interacts with CoASH to yield succinyl-CoA and dihydrolipoamide. The dihydrolipoamide is re-oxidized by the E_3_ subunit where the liberated electrons are transferred through FAD to reduce NAD^+^ forming NADH. Note that Ogdh contains two ROS generating sites depicted by red stars and can also be S-glutathionylated on all three subunits. (For interpretation of the references to color in this figure legend, the reader is referred to the web version of this article.)

It is appreciated more than ever that controlled low grade ROS formation is required to modulate cell functions in tandem with other signaling cascades [1]. Mitochondria are quantifiably the most important source of ROS using it as a superimposed co-signal to fine tune the timing of cellular signaling pathways [1], [2]. It is imperative that ROS formation is maintained under strict control since higher than normal production can irreversibly damage cell constituents. ROS levels are maintained by a suite of antioxidant systems dedicated to quenching different oxyradicals generated as a consequence of electron movement in mitochondria [3]. Mitochondrial ROS genesis starts when a small fraction of electrons liberated during nutrient oxidation prematurely exit the respiratory chain to univalently reduce O_2_ generating the proximal mitochondrial ROS superoxide ($O_{2}^{∙-}$) (Fig. 1a) [3], [4]. Superoxide is then rapidly dismutated by superoxide dismutase (SOD) yielding hydrogen peroxide (H_2_O_2_) [4]. Following its production, H_2_O_2_ has multiple fates; it is either quenched by redox buffering networks or utilized in signaling [5]. The former is composed of two key systems; the glutathione (GSH) system and thioredoxin system [6]. The GSH system is composed of two enzymes glutathione peroxidase (Gpx) and glutathione reductase (GR). For H_2_O_2_ quenching, Gpx reacts with 2 molecules of GSH in tandem with H_2_O_2_ to yield glutathione disulfide (GSSG) which is rapidly reduced back to GSH by GR in the presence of NADPH. Notably, GSH is highly concentrated in mitochondria (1–5 mM) relative to GSSG (~0.1 mM) which also imbues it with capacity to serve as a major redox buffer [7].

Redox signaling has emerged as an important means for controlling mitochondrial bioenergetics [8], [9]. Signaling is mediated through the modification of redox switches, protein cysteine thiols that sense local changes in the mitochondrial redox buffering environment [10]. Mitochondria contain a number of these switches which are found in Krebs cycle enzymes, respiratory complexes, and proteins required to drive mitochondrial fission/fusion, protein import, or induction of mitoptosis [9], [11], [12]. These redox switches also serve as an important means of modulating mitochondrial ROS formation where changes in mitochondrial redox buffering networks feedback to inhibit ROS formation at sites of production [10]. Although cysteines can undergo a range of redox modifications, mitochondria are a '*hot-bed*‘' for S-glutathionylation reactions which involves the formation of a disulfide bridge between cysteine and glutathione [13]. Mitochondria contain a number of S-glutathionylation targets which includes enzymes and respiratory complexes that generate ROS [8]. These reactions are highly specific and enzymatically mediated. Specificity is conferred by the presence of S-glutathioylation motifs, characterized by solvent accessible cysteine residues surrounded by positively charged amino acids. S-glutathionylation reactions are catalyzed by glutaredoxins (Grx), thiol oxidoreductases that harbor glutathionylase and deglutathionylase activities [14]. Grx2, which is found in the matrix of mitochondria, catalyzes the reversible S-glutathionylation of Complex I in response to changes in GSH/GSSG which modulates ROS production [15], [16]. Grx2-mediated S-glutathionylation reactions also modulate uncoupling protein-3 mediated control over mitochondrial ROS formation [17].

Ogdh sits at a major metabolic hub in the Krebs cycle linking carbon flux to the biosynthesis and degradation of amino acids. The catalytic activity of Ogdh depends on three subunits, 2-oxoglutarate decarboxylase (E_1_), dihydrolipoamide succinyltransferase (E_2_), and dihydrolipoamide dehydrogenase (E_3_) which work in tandem to transfer electrons from 2-oxoglutarate at the E_1_ subunit reducing NAD^+^ to NADH at the E_3_ subunit (Fig. 1b). The NADH generated is utilized to drive the oxidative phosphorylation of ADP producing ATP. Ogdh also serves as a mitochondrial redox sensor and is modulated by changes in ROS. The vicinal thiols on dihydrolipoamide in the E_2_ subunit are especially sensitive to oxidation by H_2_O_2_ which decreases Ogdh activity in response to increased mitochondrial ROS [18]. Following oxidation by H_2_O_2_, S-glutathionylation is required to protect vicinal thiols on lipoic acid in the E_2_ subunit from further oxidation [19]. Importantly S-glutathionylation of Ogdh is reversible where removal of GSH fully restores enzyme activity [19]. On top of its role as a redox sensor, Ogdh also produces ROS and has recently been shown to be a major source of $O_{2}^{∙-}$/H_2_O_2_ [20]. Ogdh can produce $O_{2}^{∙-}$/H_2_O_2_ when metabolizing 2-oxoglutarate and NAD^+^ or in the presence of μM amounts of NADH [20], [21]. The E_3_ subunit of Ogdh has been characterized as the chief site for $O_{2}^{∙-}$/H_2_O_2_ production but recent work has also shown that ROS can also be emitted from the E_1_ subunit [20], [22]. Pyruvate dehydrogenase (Pdh), an enzyme similar in structure to Ogdh which commits carbohydrate degradation product pyruvate to Krebs cycle metabolism, also serves as a redox sensor, increasing and decreasing ROS production in response to changes in mitochondrial redox buffering capacity [23]. Thus, Ogdh not only serves as a redox sensor but through ROS formation directly influences the state of the surrounding redox environment.

Changes in nutrient metabolism and electron supply are at the heart of cellular redox signaling [24]. It is thus critical to further characterize the redox sensing properties of enzymes like Ogdh, which provide a direct link between carbon flux and changes in redox environment. Here, we hypothesized that S-glutathionylation reactions would control ROS genesis by Ogdh. It was reasoned that a low GSH/GSSG, driven by an increase in GSSG, would spontaneously S-glutathionylate Ogdh diminishing its activity while simultaneously suppressing ROS production. Likewise a high GSH/GSSG of ~10–100 would have the opposite effect, restoring Ogdh activity while driving ROS production back up to control levels. GSSG at mM concentrations, which would only be found in mitochondria experiencing oxidative stress, led to S-glutathionylation of Ogdh lowering $O_{2}^{∙-}$/H_2_O_2_ production and 2-oxoglutarate oxidation. However, it was unexpectedly found that GSH amplified $O_{2}^{∙-}$/H_2_O_2_ production while simultaneously suppressing NADH production. In addition, Grx2 reversed these effects showing that this amplification can be reversed and is thus controlled. Collectively, our results point to the existence of a regulatory mechanism that involves the Ogdh-mediated sensing of changes in mitochondrial redox environment where a reduced glutathione pool can feed back and amplify ROS production which can be reversed by activated Grx2.

## 2 Experimental procedures

### 2.1 Reagents

Purified Ogdh from porcine heart (α-ketoglutarate dehydrogenase; Sigma Catalog Number K1502), Grx2 (human recombinant protein purified from *Escherichia coli*, Sigma Catalog Number G6673), mannitol, sucrose, EGTA, and Hepes, 2-oxoglutarate, NAD^+^, NADH, NADPH, GR, GSH, GSSG, hydroxyethyl disulfide (HEDS), thiamine pyrophosphate (TPP), Coenzyme A (CoASH), 3-methyl-2-oxopentanoic acid (KMV) and horseradish peroxidase (HRP), superoxide dismutase (SOD), catalase (CAT), alamethicin, 1-chloro-2,4-dinitrobenzene (CDNB), and defatted BSA were purchased from Sigma. Amplex UltraRed (AUR) were purchased from Invitrogen.

### 2.2 Ogdh assays

Ogdh was examined essentially as described previously with a few modifications [20], [25]. All assays were conducted in MESH buffer (220 mM mannitol, 90 mM sucrose, 20 mM Hepes, 1 mM EGTA, pH 7.4) at 25 °C. Ogdh was diluted to 20 mU/mL in MESH containing 0.3 mM TPP, and 0.1 mM CoASH and allowed to equilibrate for 5 min at 25 °C. NAD^+^ (1 mM) was then added followed by initiation of reactions with 2-oxoglutarate (final concentration range from 0.1 to 20 mM). For most assays, reactions were driven by 10 mM 2-oxoglutarate unless stated otherwise. Formation of NADH was monitored over 10 min at 30 s intervals and tracked by autofluorescence (ex:em; 376:420) using a Synergi MX2 monochromatic microplate reader (BioTek). Amount of NADH produced was quantified using standard curves and Gen5 software. To test the impact of glutathione on its activity, Ogdh was preincubated in GSH (1–10 mM), GSSG (0.01–10 mM), or a combination of GSH and GSSG for 5 min at 25 °C prior to enzyme equilibration. Note that GSH and GSSG were utilized in different combinations to examine if changes in GSH/GSSG redox potential affected Ogdh. For KMV assays Ogdh was pre-incubated for 5 min in KMV prior to conducting any assays. For Grx2 assays, immediately prior to initiating Ogdh activity reaction mixtures were supplemented with Grx2 and GSH at a final concentration of 0.5 mM and 1 mM respectively. Note that GSH is required to drive Grx2 activity. Rate calculations were based on the linear part of the curves (within first 3 min of the assay). The condition of Ogdh was examined prior to experiments by testing its activity in the absence or presence of 1 mM DTT.

### 2.3 Amplex Ultra Red (AUR) assays

Measurement of $O_{2}^{∙-}$/H_2_O_2_ was conducted as described in [25]. Ogdh was treated with GSH, GSSG or GSH+GSSG and then equilibrated in MESH as described above. Mixtures were then supplemented with 3 U/mL HRP, 25 U/mL SOD, and 5 μM AUR and reactions were started by addition of 2-oxoglutarate with NAD^+^ or with NADH (0.5–100 μM). Note that for experiments testing $O_{2}^{∙-}$/H_2_O_2_ formation during RET TPP and CoASH were excluded. $O_{2}^{∙-}$/H_2_O_2_ formation was monitored fluorometrically at ex:em 565 nm:600 nm. Specificity of AUR towards H_2_O_2_ formation was confirmed by including 10 U/mL CAT in reaction mixtures.

### 2.4 Grx2 activity measurement

Grx2 activity was examined by HEDS assay as described in [26]. HEDS was diluted to 1.8 mM in MESH containing 0.9 mM GSH and allowed to incubate at 25 °C for 30 min. Reaction mixtures were then supplemented with 0.5 μM Grx2 and 0.4 U/mL GR and then allowed to equilibrate for 5 min. Reactions were initiated by the addition of 1 mM GSH followed by 0.2 mM NADPH. Reactions were monitored over 5 min with measurements taken at 30 s intervals. NADPH consumption was tracked by autofluorescence (em:ex, 376 nm:450 nm). To ascertain if (1) Ogdh could undergo S-glutathionylation by GSH and (2) if Ogdh was a substrate for Grx2, Ogdh was incubated in 5 mM GSH for 5 min at 25 °C followed by the addition of Grx2, GSH, GR, and NADPH.

### 2.5 Preparation of liver mitochondria

All animal experiments were approved by Memorial University's Animal Care and Use committee. All steps were performed on ice or 4 °C unless stated otherwise. Mitochondria were enriched from the livers of male C57BL/6N mice purchased from Charles River Laboratories. Mice (9–10 weeks old) were euthanized by cerebral dislocation under isoflurane anesthesia, livers were placed in MESH containing 0.5% defatted BSA (MESH-B). Livers were cut into small pieces, washed several times to remove excess blood and then homogenized on ice in MESH-B using the Potter–Elvejham method. Homogenates were centrifuged at 800×*g* for 9 min after which fat was carefully skimmed off the top of the supernatant. The supernatant was collected, centrifuged at 10,000×*g* for 9 min to pellet mitochondria, and then decanted. Sides of the tubes were wiped carefully to remove any access fat. Mitochondrial pellets were then resuspended in 10 mL of MESH-B and centrifuged at 10,000×*g* for 9 min. The final mitochondrial pellet was resuspended in ice cold MESH and stored on ice for assays. Protein content was quantified by Bradford Assay.

Mitochondria were diluted to 5 mg/mL in pre-warmed MESH-B containing 10 μM CDNB. Suspensions were vortexed vigorously and then incubated for 10 min at room temperature. Previous studies have established that 10 μM CDNB is adequate to deplete mitochondria of GSH [25]. Suspensions were then centrifuged for 5 min at 10,000×*g* and washed twice with ice-cold MESH-B to remove any excess CDNB. Mitochondria were then resuspended in 1 mL MESH-B containing 40 μg/mL alamethicin and incubated for 5 min at 37 °C to permeabilize mitochondria [20]. Suspensions were diluted by 2.5-fold in ice-cold MESH-B and centrifuged for 15 min at 30,000×*g*. Permeabilized mitochondrial pellets were then resuspended in MESH-B at a final concentration of 3 mg/mL for assays. Efficacy of permeabilization was tested by measuring the activity of malate dehydrogenase (Mdh) [27]. Ogdh activity and $O_{2}^{∙-}$/H_2_O_2_ production and the effects of GSH and Grx2 were examined essentially as described above. Assays were conducted on permeabilized mitochondria diluted to a final concentration of 0.3 mg/mL.

### 2.6 Mitochondrial GSH levels

Mitochondrial glutathione levels were determined using 5,5′-dithio-bis(2-nitro-benzoic acid; DTNB). Briefly mitochondria were treated with or without CDNB and then permeabilized as described above followed by incubation in 5 mM GSH for 5 min at 25 °C. Mitochondria were then pelleted and resuspended at a final concentration of 5 mg/mL in 10% trichloroacetic acid solution and then allowed to incubate on ice for 10 min. Precipitated protein was pelleted by high speed centrifugation and the supernatant was collected and stored on ice. The pH was neutralized with 6 N KOH and then diluted 2-fold with pre-warmed MESH containing 0.2 mM DTNB. Reactions were then incubated at 37 °C for 30 min. Color changes were monitored at *A*_412_. GSH levels were quantified using standard curves with a GSH concentration range of 0.1–10 mM.

### 2.7 Immunoblotting

Purified Ogdh was diluted to 2 mg/mL in MESH and incubated for 10 min at 25 °C in 1 or 10 mM GSSG, or 5 mM GSH in a final volume of 150 μL. For reactions involving Grx2, following the 15 min incubation, samples were then incubated for an additional 5 min in 1 µM Grx2+2 mM GSH. Reactions were then stopped by adding 1 volume of RIPA buffer containing 25 mM N-ethylmaleimide (NEM). After a 5 min incubation at room temperature reaction mixtures were diluted with a volume of sample buffer with or without 2% (v/v) 2-mercaptoethanol. The final concentration of Ogdh was 0.5 mg/mL and 25 µL of sample was loaded per well giving a final amount of 12 µg of protein. Anti-Ogdh(Abcam) was used at a final dilution of 1:5000 and protein-glutathione mixed disulfide (PSSG) anti-serum (Virogen) was diluted to 1:1000. Primary antibodies were prepared in TBS-T containing 5% (w/v) fatty acid-free BSA and 0.02% (w/v) sodium azide. Secondary antibodies were horse radish peroxidase conjugated goat anti-rabbit and goat anti-mouse (1:3000), respectively. Proteins were detected using Supersignal West Pico (Thermo Scientific) and bands were visualized with ImageQuant LAS 4000 (GE Health Care Life Sciences).

### 2.8 Data analysis

For purified enzyme assays all experiments were performed 4 times and in duplicate. Permeabilized mitochondria experiments were performed 6 times and in quadruplicate. Western blots were performed in duplicate except for blots in Fig. 6d which were performed 4 times and then quantified for band intensity with ImageJ software. All results were analyzed using GraphPad Prism 6 software.

## 3 Results

### 3.1 GSSG lowers $O_{2}^{∙-}$/H_2_O_2_ formation by purified Ogdh

**Fig. 2:**
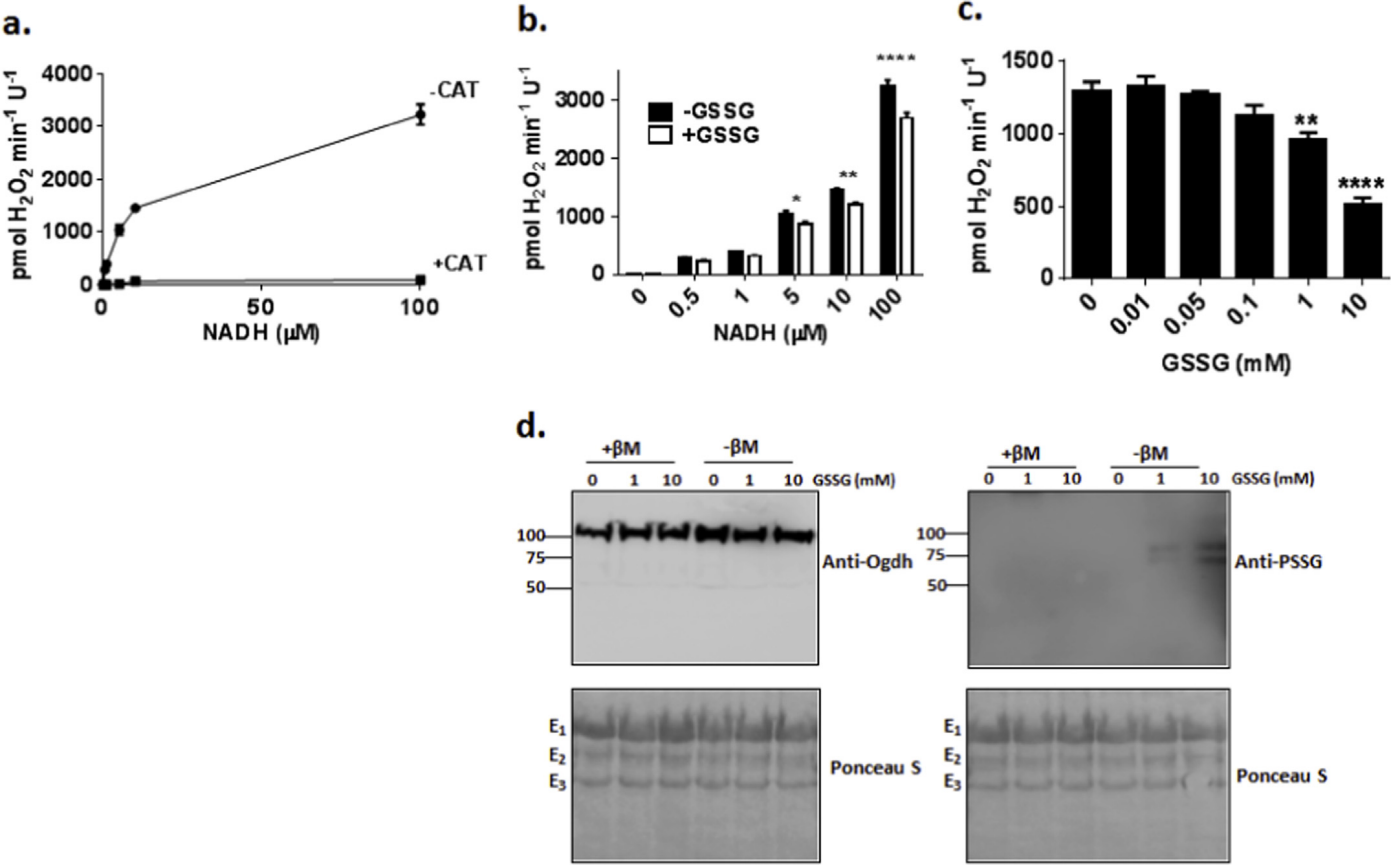
GSSG mildly suppresses $O_{2}^{∙-}$/H_2_O_2_ through S-glutathionylation of its E_2_ subunit. (A) Purified Ogdh complex generates $O_{2}^{∙-}$/H_2_O_2_ from reverse electron transfer from NADH. Purified Ogdh was treated with different concentrations of NADH (0–100 μM) and then $O_{2}^{∙-}$/H_2_O_2_ production was measured using Amplex Ultra Red (AUR). Catalase (CAT) was included (10 U/mL) as a control to ensure AUR was specific for H_2_O_2_. (B) Impact of GSSG on $O_{2}^{∙-}$/H_2_O_2_ production from purified Ogdh complex oxidizing NADH. Reactions were conducted as in A except Ogdh was pre-incubated in 1 mM GSSG prior to initiating reactions. *n*=4, 2-way ANOVA with Tukey's post-hoc test. (C) Effect of different GSSG concentrations on $O_{2}^{∙-}$/H_2_O_2_ formation by reverse electron flow. Purified Ogdh was pre-incubated in GSSG (0–10 mM) and then $O_{2}^{∙-}$/H_2_O_2_ production was examined by AUR using 5 microM NADH as the substrate. *n*=4, 1-way ANOVA with Tukey's post-hoc test. (D) Examination of GSSG-mediated S-glutathionylation of Ogdh by immunoblot. Protein glutathione mixed disulfide (PSSG) formation was examined using anti-PSSG antibody. Specificity of anti-PSSG towards glutathionylated protein was assured by running gels under reducing conditions (+βM; 2% β-Mercaptoethanol added to sample buffer). E_1_ subunit (~110 kDa) was detected using anti-E_1_ antibody. All three subunits were detected by Ponceau S staining of blots. Stains were documented and individual molecular weights for each subunit were estimated using a molecular weight marker and ImageQuant software.

Previous studies have shown that Ogdh can undergo S-glutathionylation which can limit its activity [28]. In particular it was found that S-glutathionylation was potentiated by oxidation of vicinal lipoamide thiols on the E_2_ subunit of Ogdh resulting its S-glutathionylation which is required to protect the enzyme complex from irreversible deactivation [28]. However, it has also been found that GSSG at mM concentrations can also drive S-glutationylation of Ogdh on all three subunits [29]. To ascertain if Ogdh was (1) sensitive to S-glutathionylation by GSSG and (2) if GSSG induced S-glutathionylation modulates $O_{2}^{∙-}$/H_2_O_2_ formation by Ogdh, a series of experiments were carried out to examine if S-glutathionylation can control ROS production by Ogdh. The capacity of purified Ogdh to generate $O_{2}^{∙-}$/H_2_O_2_ by RET from NADH was first confirmed Fig. 2a. A concentration dependent increase in $O_{2}^{∙-}$/H_2_O_2_ formation was observed with increasing NADH concentration (Fig. 2a). Specificity of Amplex Ultra Red for H_2_O_2_ was confirmed with catalase (CAT) which completely abolished any fluorescence. Incubation in 1 mM GSSG suppressed $O_{2}^{∙-}$/H_2_O_2_ formation by purified Ogdh in the presence of 5–100 µM NADH (Fig. 2b). Exposure of purified Ogdh to varying concentrations of GSSG revealed that at least 1 mM GSSG was required to significantly suppress NADH-driven $O_{2}^{∙-}$/H_2_O_2_ formation (Fig. 2c). $O_{2}^{∙-}$/H_2_O_2_ production decreased further (~2.5 fold) in the presence of 10 mM GSSG (Fig. 2c). Next, we utilized anti-serum directed against PSSG adducts to ascertain if these effects were associated with S-glutathionylation of Ogdh. As shown in Fig. 2d, incubation of Ogdh in GSSG caused a concentration dependent increase in an immunoreactive band at between 65 and 75 kDa which corresponded to the molecular weight of the E_2_ subunit of Ogdh. Inclusion of β-mercaptoethanol confirmed the specificity of anti-PSSG antibody towards S-glutathionylation adducts. These results confirm that GSSG can S-glutathionylate Ogdh through a simple disulfide exchange reaction.

**Fig. 3:**
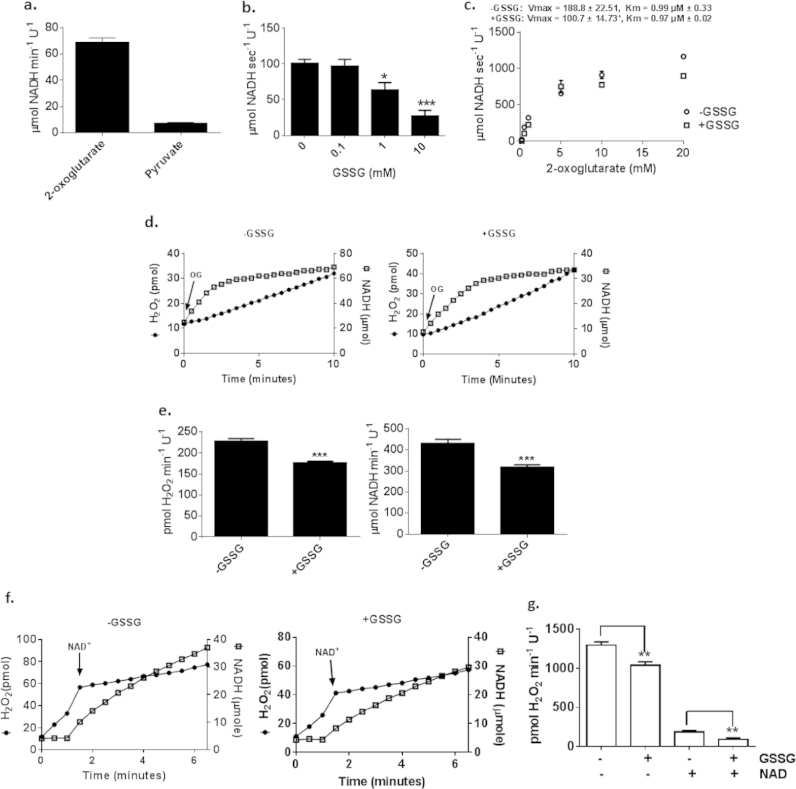
GSSG lowers the rate of $O_{2}^{∙-}$/H_2_O_2_ by Ogdh when oxidizing 2-oxoglutarate with NAD^+^. (A) Assessment of Ogdh purity. Activity of Ogdh and Pdh were examined as described in Materials and Methods except 1 mM 2-oxoglutarate or pyruvate were used to assess overall activity. (B) GSSG decreases the activity of Ogdh. Purified Ogdh was incubated in varying amounts of GSSG (0–10 mM) and then the activity examined in the presence of TPP (0.3 mM), CoASH (0.1 mM), NAD^+^ (1 mM), and 2-oxoglutarate (10 mM). (C) Effect of GSSG on Ogdh kinetics. After pre-incubating Ogdh in 1 mM GSSG rate of NADH production was measured in response to varying amounts of 2-oxoglutarate. *K*_m_ and *V*_max_ values with respect to NADH formation were calculated using Graphpad Prism software. (D) Representative traces for the simultaneous measurement of the effect of 1 mM GSSG on $O_{2}^{∙-}$/H_2_O_2_ and NADH production. Reactions were initiated by the addition of 2-oxoglutarate (OG; 10 mM). Traces were then utilized to calculate the rate of $O_{2}^{∙-}$/H_2_O_2_ and NADH production (E). (F) Traces measuring the effect of GSSG on $O_{2}^{∙-}$/H_2_O_2_ and NADH production production by Ogdh as a function of the absence and presence of NAD^+^. Reactions were initiated by the addition of 2-oxoglutarate without any NAD^+^, measured for 3 min, and then NAD^+^ was added to a final concentration of 1 mM. Traces were utilized to calculate rate of $O_{2}^{∙-}$/H_2_O_2_ and NADH production before and after addition of NAD^+^ (G). *n*=4, 1-way ANOVA with Tukey's post-hoc test.

It has been demonstrated that Ogdh also generates $O_{2}^{∙-}$/H_2_O_2_ when oxidizing 2-oxoglutarate in the presence of NAD^+^ [20]. Thus we set out to examine the effect of GSSG on Ogdh kinetics and if GSSG could alter $O_{2}^{∙-}$/H_2_O_2_ production when Ogdh is oxidizing 2-oxoglutarate in the presence of NAD^+^. Purified Ogdh from Sigma is reported to have contaminating levels of pyruvate dehydrogenase (Pdh). Thus, the activities of Ogdh and Pdh were examined in the purified Ogdh mixture. As shown in Fig. 3a, Ogdh makes up ~90% of the total activity of the preparation with Pdh making up the remaining ~10%. With this it is expected that contaminating Pdh will interfere minimally with these assays, especially since reactions are being driven by 2-oxoglutarate. GSSG at 1 mM was required to significantly lower Ogdh activity which decreased further when exposed to 10 mM GSSG (Fig. 3b). Furthermore, exposure to 1 mM GSSG decreased the *V*_max_ for 2-oxoglutarate (Fig. 3c). No change in *K*_m_ for 2-oxoglutarate was found indicating GSSG is a non-competitive inhibitor. To assess the relationship between NADH formation and $O_{2}^{∙-}$/H_2_O_2_ genesis in the presence of GSSG, both NADH and $O_{2}^{∙-}$/H_2_O_2_ production were monitored simultaneously. For these assays, the concentration of NAD^+^ was kept constant at 0.1 mM (Fig. 3d and e). Addition of 2-oxoglutarate resulted in the rapid conversion of NAD^+^ to NADH gradually reaching steady state at 2–3 min (Fig. 3d). This was associated with a linear increase in H_2_O_2_ production over 10 min. This in contrast to results collected with pyruvate dehydrogenase (Pdh) where $O_{2}^{∙-}$/H_2_O_2_ formation was reported to be directly proportional to NADH levels [25]. Pre-incubation of Ogdh with 1 mM GSSG induced a small but significant decrease in both the rate of NADH and $O_{2}^{∙-}$/H_2_O_2_ production (Fig. 3d and e). When GSSG was included in reaction mixtures NADH production began to level off later reaching steady state at ~4 min (Fig. 3d and e). To examine the effect of GSSG on Ogdh kinetics further, NADH and $O_{2}^{∙-}$/H_2_O_2_ production was measured simultaneously before and after addition of NAD^+^ (Fig. 3f and g). As noted in Fig. 3f, rates of NADH formation were negligible the absence of NAD^+^ which was associated with a rapid production of $O_{2}^{∙-}$/H_2_O_2_. Addition of NAD^+^ to reaction mixtures suppressed $O_{2}^{∙-}$/H_2_O_2_ production, which coincided with a robust increase in NADH formation (Fig. 3f). Thus, in the absence of NAD^+^ Ogdh generates high amounts of $O_{2}^{∙-}$/H_2_O_2_ due to the diversion of electrons from 2-oxoglutarate towards the univalent reduction of O_2_ and the production of ROS. Pre-incubation of Ogdh in 1 mM GSSG induced a significant decrease in $O_{2}^{∙-}$/H_2_O_2_ production before and after the addition of 0.1 mM NAD demonstrating that S-glutathionylation by GSSG can induce a mild but significant decrease in ROS production when Ogdh is oxidizing 2-oxoglutarate (Fig. 3g). Considering that GSSG is typically ~0.1 mM it can be assumed that GSSG-mediated S-glutathionylation of Ogdh may only occur in mitochondria experiencing oxidative stress (*e.g.* there is sufficient ROS accumulation to prompt an increase in GSSG to the mM range) [14]. Taken together, these results indicate that GSSG at mM concentrations can weakly react with Ogdh to induce S-glutathionylation on the E_2_ subunit of Ogdh which mildly suppresses $O_{2}^{∙-}$/H_2_O_2_ formation with a concomitant decrease in Ogdh activity.

### 3.2 GSH amplifies $O_{2}^{∙-}$/H_2_O_2_ by Ogdh

**Fig. 4:**
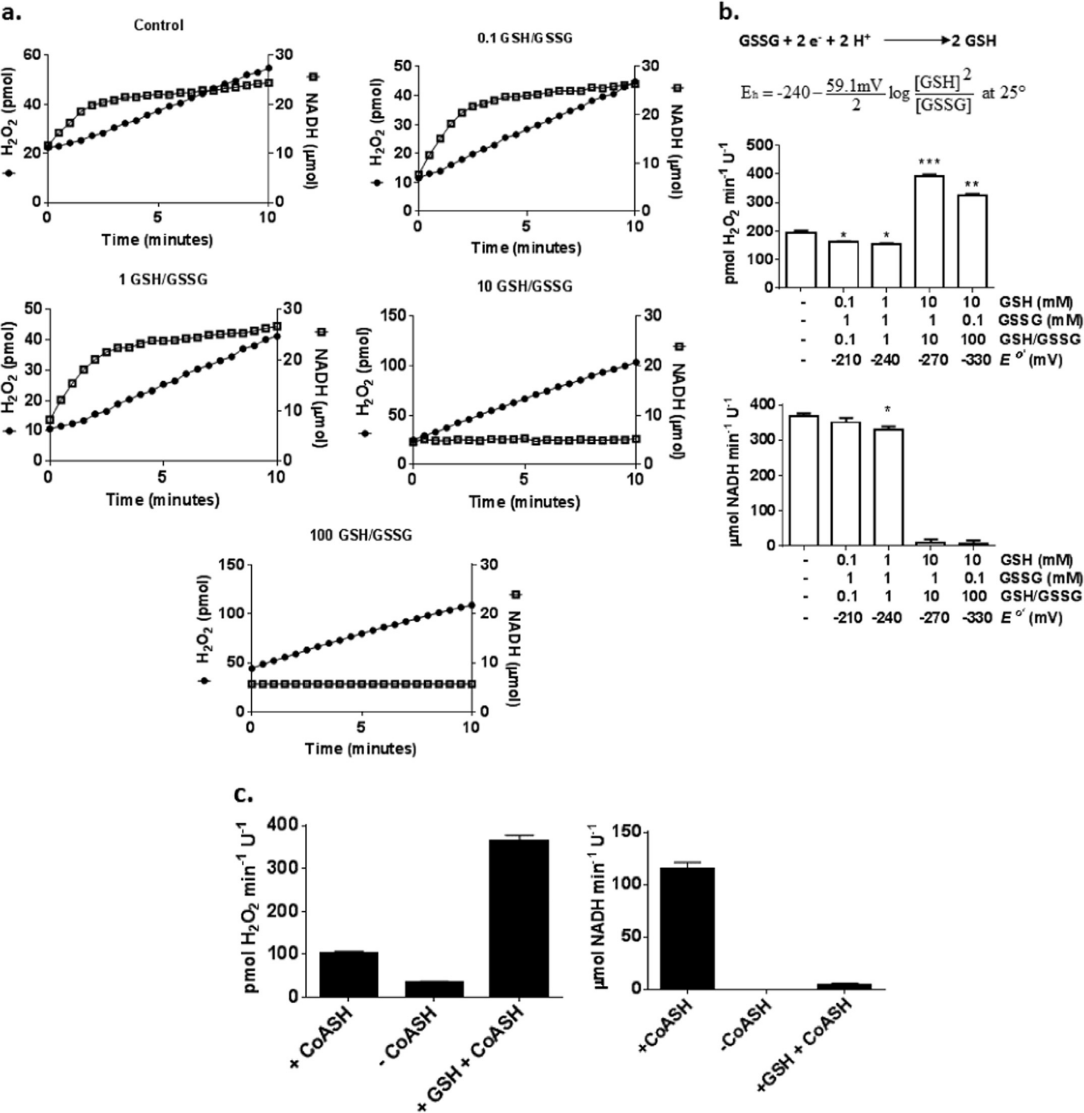
Effect of varying GSH/GSSG on Ogdh function. (A) Simultaneous examination of the effect of different GSH/GSSG ratios on $O_{2}^{∙-}$/H_2_O_2_ and NADH production by purified Ogdh. (B) Traces from A were utilized to calculate the rate of $O_{2}^{∙-}$/H_2_O_2_ and NADH production. GSH/GSSG ratios were achieved by varying the final concentrations of GSH and GSSG in reaction mixtures. The redox potential (*E*_h_) obtained in the reaction mixtures was calculated using the Nernst equation. (C) Effects of GSH on Ogdh are not due to glutathione-mediated depletion of CoASH. *n*=4, 1-way ANOVA with Tukey's post-hoc test.

Since the mitochondrial matrix usually contains both GSH and GSSG which can vary in concentration creating highly localized redox gradients we decided to examine the effects of different GSH/GSSG ratios on purified Ogdh kinetics. We postulated that while a low GSH/GSSG would impede Ogdh function limiting $O_{2}^{∙-}$/H_2_O_2_ formation, a high GSH/GSSG would have the opposite effect restoring Ogdh function back to control levels. For this set of experiments, Ogdh was pre-incubated in different concentrations of GSH and GSSG clamping GSH/GSSG at 0.1, 1, 10 and 100 (Fig. 4a and b). The calculated redox potentials for each glutathione pair are shown in Fig. 4b. After the pre-incubation, reactions were initiated by addition of 2-oxoglutarate followed by simultaneous measurement of NADH and $O_{2}^{∙-}$/H_2_O_2_ formation (Fig. 4a). We found that the presence of high GSSG (1 mM) and low GSH (0.1 and 1 mM), which clamped the GSH/GSSG at 0.1 and 1, respectively, induced a small but significant decrease in $O_{2}^{∙-}$/H_2_O_2_ production (Fig. 4a and b). At a GSH/GSSG ratio of 1, this was also associated with a small but significant decrease in Ogdh activity. However, contrary to our hypothesis we found that high GSH/GSSG (10 and 100) completely abolished NADH formation (Fig. 4a and b). This change in Ogdh function coincided with an increase in $O_{2}^{∙-}$/H_2_O_2_ production (Fig. 4a and b). CoASH can form adducts with GSH which could interfere with assay conditions which could be the reason for the increased production of ROS by Ogdh when pre-incubated with GSH. We thus decided to test the effect of the absence of CoASH on $O_{2}^{∙-}$/H_2_O_2_ production. Exclusion of CoASH induced ~70% drop in $O_{2}^{∙-}$/H_2_O_2_ production formation by the complex (Fig. 4c). Notably Ogdh still retained some ROS forming capacity which is likely associated with production by the E_1_ subunit. Also exclusion of CoASH abolished Ogdh activity. By way of contrast pre-incubation of Ogdh with GSH at a final concentration of 5 mM induced a robust ~3.5-fold increase in ROS production (Fig. 4c). This was also associated with a loss in Ogdh activity.

**Fig. 5:**
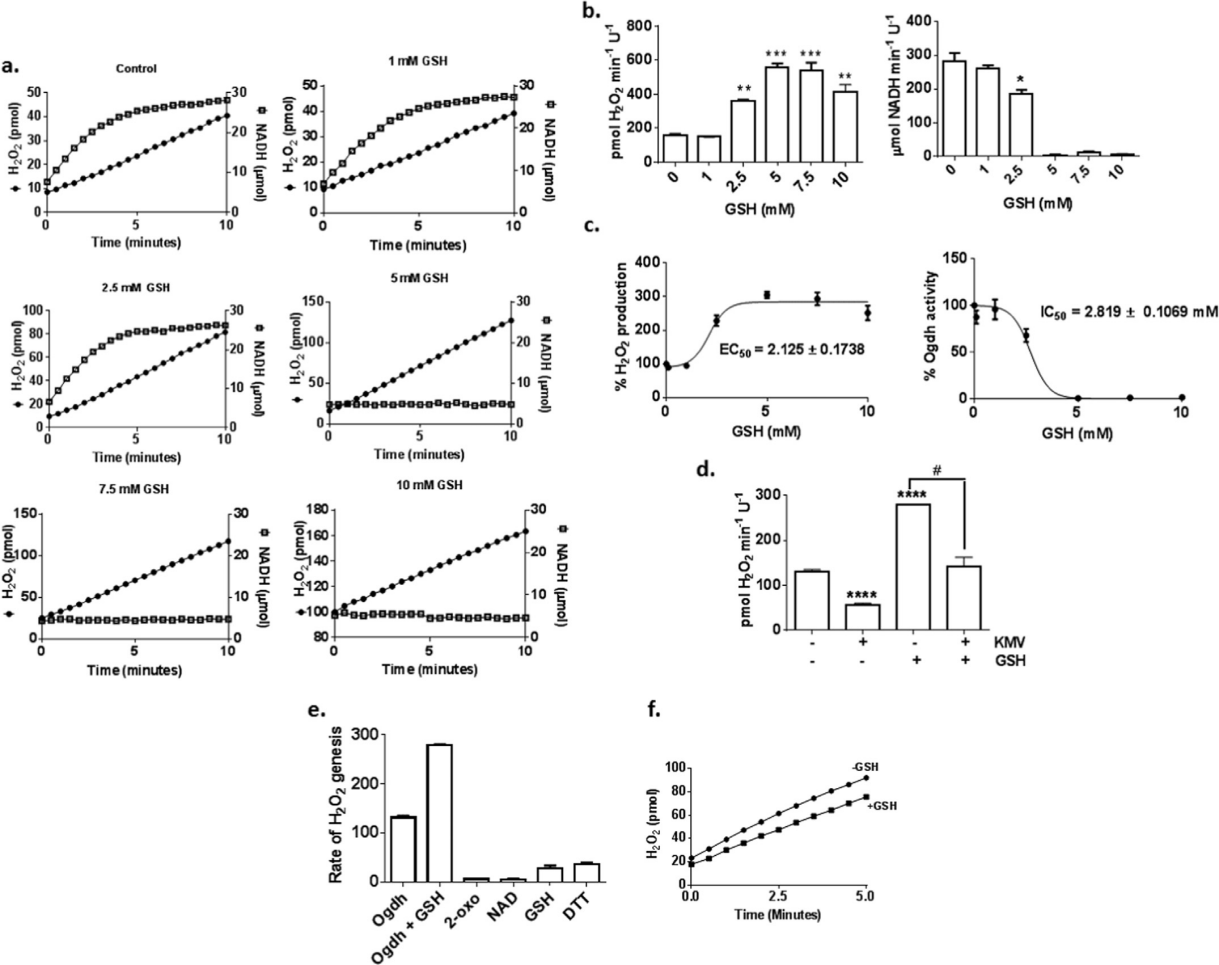
GSH alters electron flux in purified Ogdh resulting in amplified $O_{2}^{∙-}$/H_2_O_2_ production. (A) Representative traces showing the effect of different GSH concentrations (0–10 mM) on $O_{2}^{∙-}$/H_2_O_2_ and NADH production. Note that GSH≥5 mM completely abolishes NADH formation. Traces were utilized to calculate rates of $O_{2}^{∙-}$/H_2_O_2_ and NADH production (B). (C) Dose–response curves for the effect of GSH on Ogdh activity and $O_{2}^{∙-}$/H_2_O_2_ formation. (D) Impact of Ogdh inhibitor 3-methyl-2-oxopentanoic acid (KMV) on $O_{2}^{∙-}$/H_2_O_2_ and NADH production in the presence or absence of 5 mM GSH. (E) Autooxidation of AUR by different reaction mixture components. Calculated rates $O_{2}^{∙-}$/H_2_O_2_ production were compared to reactions containing Ogdh and Ogdh+5 mM GSH. Note that GSH (5 mM) and dithiol DTT (5 mM) induce a small change in the rate of AUR conversion to resorufin. (F) GSH has little effect on the rate of $O_{2}^{∙-}$/H_2_O_2_ production during reverse electron transfer from NADH. Reactions were initiated by the addition of NADH (5 µM) and then the reaction of AUR with H_2_O_2_ was measured. *n*=4, 1-way ANOVA with Tukey's post-hoc test.

The unexpected nature of these findings prompted us to examine the impact of GSH on purified Ogdh further. In this next set of experiments, Ogdh was treated with different concentrations of GSH to pinpoint the concentration required to induce an increase in ROS production while simultaneously suppressing its activity. As shown in Fig. 5a and b, pre-incubation of Ogdh in increasing GSH concentrations led to a progressive augmentation of $O_{2}^{∙-}$/H_2_O_2_ formation reaching a peak of 3.5-fold at 5 mM GSH. The lowest concentration of GSH required to induce this effect was 2.5 mM GSH which increased $O_{2}^{∙-}$/H_2_O_2_ formation by ~2.4-fold (Fig. 5a and b). This effect was associated with a significant decrease in NADH formation (~33% decrease in activity). Concentrations of GSH≥5 mM completely abolished NADH production amplifying $O_{2}^{∙-}$/H_2_O_2_ production further. Thus, when exposed to 2.5 mM GSH Ogdh retains ~67% of its activity which is associated with a ~2.4-fold increase in ROS production. These findings prompted us to construct dose response curves to pinpoint the concentration of GSH required to induce a 50% increase in ROS formation and a 50% decrease in Ogdh activity. Dose response curve calculations revealed that 2.125 mM GSH was required to induce a 50% increase in $O_{2}^{∙-}$/H_2_O_2_ formation by Ogdh while 2.819 mM was required to induce a 50% decrease in activity (Fig. 5c). Considering that concentrations of GSH in mitochondria are found between 1 and 5 mM we can conclude that physiologically relevant concentrations of GSH can induce significant changes in Ogdh activity and $O_{2}^{∙-}$/H_2_O_2_ formation [7], [15]. Also, considering ROS production and redox buffering are also in a constant state of flux it can be presumed that if GSH concentrations dip below 2 mM, Ogdh can retain a higher NADH and lower ROS production profile [24].

To confirm that GSH was amplifying ROS production reactions were performed in the presence of Ogdh inhibitor KMV. KMV impedes Ogdh activity by blocking the 2-oxoglutarate binding site on the E_1_ subunit. Inclusion of KMV induced a decrease $O_{2}^{∙-}$/H_2_O_2_ formation in control reactions confirming that this compound is able to inhibit ROS formation via blockage of the 2-oxoglutarate binding site of Ogdh (Fig. 5d). Note that it has been shown that KMV does not completely inhibit Ogdh activity [20]. Incubation of Ogdh in 5 mM GSH led to a robust increase in $O_{2}^{∙-}$/H_2_O_2_ production which was prevented by KMV (Fig. 5d). It has been reported GSH can autooxidize Amplex Red generating a fluorescence comparable to respiring mitochondria [30]. Thus we tested if GSH, 2-oxoglutarate, NAD^+^ or dithiol DTT could interfere with Amplex Red. GSH and DTT induced a small increase in detectable fluorescence but this was significantly surpassed by the rate of production by Ogdh in the absence or presence of GSH (Fig. 5e). The impact of GSH on NADH-driven $O_{2}^{∙-}$/H_2_O_2_ formation was also measured (Fig. 5f). We found that GSH induced a small decrease in $O_{2}^{∙-}$/H_2_O_2_ production during RET indicating GSH impedes Ogdh upstream of the E_3_ subunit. Collectively, these results indicate that the concentrations of GSH normally found in mitochondria amplifies $O_{2}^{∙-}$/H_2_O_2_ production by Ogdh by redirecting electron flow from NADH formation to ROS genesis upstream of the E_3_ subunit.

### 3.3 Ogdh is a target for Grx2-mediated protein deglutathionylation

**Fig. 6:**
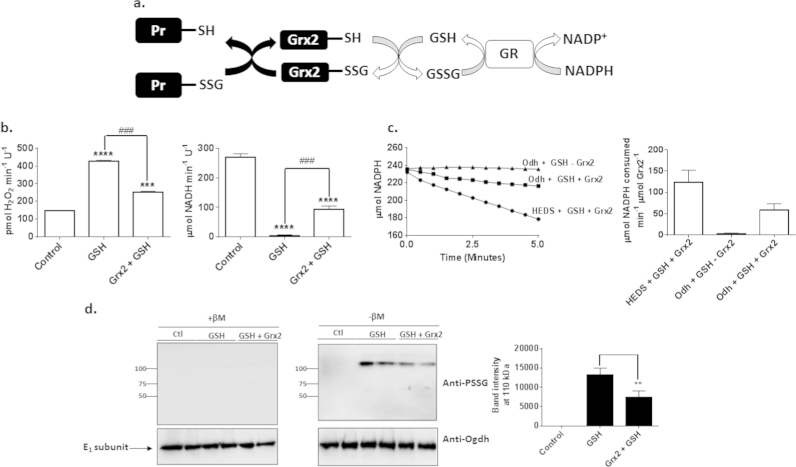
Glutaredoxin-2 (Grx2) targets Ogdh for deglutathionylation reversing GSH-mediated amplification of $O_{2}^{∙-}$/H_2_O_2_ formation. (A) Schematic depiction of the Grx2-mediated deglutathionylation of target proteins. Successful deglutathionylation requires GSH which is utilized to finalize the catalytic cycle of Grx2. (B) Grx2 reverses the GSH-mediated amplification and $O_{2}^{∙-}$/H_2_O_2_ formation and abolishment of NADH production. Purified Ogdh was pre-incubated with or without GSH (5 mM) and then assayed for $O_{2}^{∙-}$/H_2_O_2_ and NADH production. Purified Grx2 (0.5 µM) was added with GSH (1 mM) immediately before starting measurements. Reactions were initiated by the addition of 2-oxoglutarate (10 mM). (C) Ogdh is a substrate for Grx2. Grx2 activity was assayed as described in Materials and Methods. To ensure Grx2 was enzymatically active, its activity was measured using the hydroethyl disulfide (HEDS) assay. To test if Ogdh is a target for Grx2, Ogdh was preincubated in GSH (5 mM) and then Grx2 (0.5 µM) was added in combination with GSH, GR, and NADPH. Grx2 activity is associated with the consumption of NADPH. Representative traces were utilized to calculate the rate of NADPH consumption (D). (D) Assessment of protein glutathione mixed disulfide (PSSG) formation by GSH. Ogdh was incubated in 5 mM GSH and then with or without Grx2+1 mM GSH and then examined for PSSG levels. Following this membranes were stripped and reprobed for Ogdh E_1_ subunit as a loading control. Immunoreactivity of PSSG towards its anti-serum was confirmed be performing electrophoresis under reducing conditions (+βM; 2% β-Mercaptoethanol added to sample buffer). PSSG immunoreactive bands were quantified using ImageJ software. *n*=4, 1-way ANOVA with Tukey's post-hoc test.

Grx2 is responsible for deglutathionylating target proteins in mitochondria in the presence of GSH ( Fig. 6a). Following a round of deglutathionylation GSSG is reduced in the presence of NADPH and GR. Based on this experiments were conducted to determine if Grx2 at concentrations found in mitochondria (~0.5 μM) could reverse the GSH mediated amplification of $O_{2}^{∙-}$/H_2_O_2_ production. Pre-incubation of purified Ogdh in 5 mM GSH enhanced $O_{2}^{∙-}$/H_2_O_2_ with the concomitant abolishment of NADH formation (Fig. 6b). Addition of Grx2 with 1 mM GSH partially recovered Ogdh activity which was associated with a concomitant decrease in ROS production (Fig. 6b). We next sought to ascertain if Ogdh pre-incubated in GSH could serve as a substrate for Grx2. Enzymatic activity of Grx2 operates in three stages; (1) catalytic N-terminal cysteine of Grx2 displaces the glutathionyl moiety from protein glutathione mixed disulfide (PSSG) resulting in the formation of a glutathionyl-Grx2 (Grx2-SG) intermediate, (2) GSH binds Grx2-SG resulting in the deglutathionylation of Grx2 and the formation of GSSG, and (3) GSSG is oxidized by glutathione reductase (GR) in the presence of NADPH to yield 2 GSH (Fig. 6a) [31]. To test if Grx2 could deglutathionylate Ogdh we utilized a modified hydroxyethyldisulfide (HEDS) assay where Ogdh pre-incubated in 5 mM GSH served as the Grx2 substrate. The HEDS assay revealed that Grx2 was enzymatically active (Fig. 6c). Reactions in the absence of Grx2 showed that NADPH was not spontaneously oxidized (Fig. 6c). Inclusion of Grx2 in reaction mixtures containing Ogdh pre-incubated in GSH stimulated the GR-mediated consumption of NADPH at almost half the rate observed with the HEDS assay (Fig. 6c). This result would suggest that Ogdh pre-incubated in GSH could serve as a substrate for Grx2. Next, immunoblot was conducted to determine if GSH could modify Ogdh by S-glutathionylation. As shown in Fig. 6d, GSH induced a robust increase in the presence of an immunoreactive band at ~110 kDa which would correspond to the E_1_ subunit of purified Ogdh. Reactions that included Grx2 with 1 mM GSH lowered the intensity of this immunoreactive band (Fig. 6d). This would indicate that GSH and GSSG have different S-glutathionylation targets in Ogdh. Inclusion of 2% β-mercaptoethanol in the preparations abolished the immunoreactivity of this band towards anti-PSSG (Fig. 6d). These results illustrate that GSH amplifies ROS formation by Ogdh by blocking electron flow through modification of the E_1_ subunit. These effects are reversed by the Grx2-mediated deglutathionylation of Ogdh.

### 3.4 GSH amplifies $O_{2}^{∙-}$/H_2_O_2_ production by Ogdh in liver mitochondria which is reversed by Grx2

**Fig. 7:**
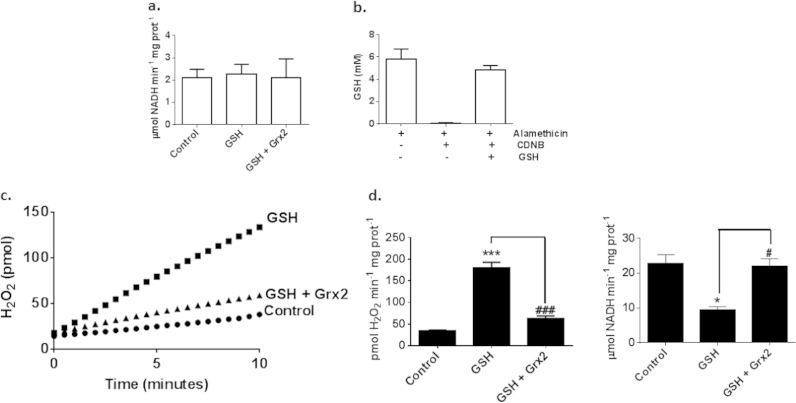
Result of GSH and Grx2 on Ogdh-mediated $O_{2}^{∙-}$/H_2_O_2_ in liver mitochondria. (A) Effectiveness of alamethicin in permeabilizing mitochondria was tested by measuring malate dehydrogenase (Mdh) activity. Mitochondria were depleted of GSH by CDNB, permeabilized, and then re-supplemented with GSH (5 mM) or Grx2 (0.5 µM)+1 mM GSH. Reaction mixtures consisted of NAD^+^ (1 mM) and measurements were initiated following addition of malate. (B) CDNB depletes mitochondrial glutathione. Mitochondria were treated with or without CDNB (10 µM), permeabilized with alamethicin (40 µg/mL). Glutathione levels were estimated using Ellman's reagent. To ensure that GSH added to reaction mixtures could penetrate the matrix, permeabilized mitochondria pre-incubated in CDNB were re-supplemented with 5 mM GSH. (C and D) Exogenously added GSH amplifies $O_{2}^{∙-}$/H_2_O_2_ production by Ogdh which is reversed by glutaredoxin-2 (Grx2). Incubations were performed as described in A after which $O_{2}^{∙-}$/H_2_O_2_ genesis and NADH production were examined. *n*=6, 1-way ANOVA with Tukey's post-hoc test.

The results above show that purified Ogdh derived from porcine heart can be S-glutathionylated when incubated for short periods with physiologically relevant concentrations of GSH. This is correlated with a burst in ROS production which was associated with a decrease or complete loss in Ogdh activity, which was highly dependent on GSH concentration. These effects could be reversed by Grx2, a thiol oxidoreductase known to catalyze deglutathionylation of proteins. Next, we tested whether GSH could elicit the same effects on mitochondria isolated from mouse liver. Mitochondria contain 1–5 mM GSH and are impermeable to most of the reagents used in this study. Thus, prior to conducting experiments, mitochondria were depleted of endogenous GSH using CDNB, which takes advantage of the catalytic action of endogenous glutathione S-transferase to irreversibly conjugate CDNB to GSH [32]. CDNB is utilized routinely to deplete mitochondrial GSH [23], [25] but it is worthy to point out that CDNB does interfere with other antioxidant enzymes like thioredoxin reductase but this does not preclude its use to study the effects of GSH manipulations on Ogdh activity [32]. After CDNB treatment to ensure any residual CDNB was removed mitochondria were washed and then permeabilized with alamethicin to facilitate matrix uptake of Ogdh substrates and manipulation of matrix GSH. Mitochondrial permeability was confirmed by measuring malate dehydrogenase activity which requires NAD^+^ to drive the formation of oxaloacetate (Fig. 7a). We also confirmed (1) that CDNB depleted endogenous GSH and (2) exogenously added GSH could gain access to the matrix environment (Fig. 7b). These control experiments confirm that CDNB is effective at depleting GSH and that after depletion GSH levels can be experimentally manipulated in permeabilized mitochondria.

Pre-incubation of mitochondria with 5 mM GSH induced a substantial increase in $O_{2}^{∙-}/$H_2_O_2_ formation which was associated with a decrease in NADH production (Fig. 7c and d). However, unlike purified Ogdh, NADH formation was not completely abolished which may be due to the fact that other NADH and NADPH producing enzymes are still active in the matrix (Fig. 7c and d). Adding enzymatically active Grx2 to reaction mixtures almost completely restored Ogdh-mediated NADH formation with a concomitant suppression of $O_{2}^{∙-}$/H_2_O_2_ formation (Fig. 7c and d). These results confirm that 5 mM GSH can divert electrons away from NADH production and towards ROS biosynthesis in mitochondria. It is also important to note that mitochondria from liver tissue also harbor endogenous Grx2 however; it is usually maintained in an inactive state through formation of Grx2 dimers through Fe–S chelation [33]. Indeed, Grx2 is only activated by Fe–S disassembly which is mediated by $O_{2}^{∙-}$/H_2_O_2_ resulting in the release of enzymatically active Grx2 monomers [33].

## 4 Discussion

Nutrient metabolism is at the root of redox signaling. Electrons supplied by the combustion of carbon in mitochondria generate NADH and NADPH in conjunction with $O_{2}^{∙-}$/H_2_O_2_, factors that alter mitochondrial redox buffering networks in space and time conveying redox signals to the cell [24]. Mitochondria can contain up to 12 ROS generating sites with Ogdh serving as a major source of $O_{2}^{∙-}$/H_2_O_2_ (Fig. 1a) [3], [34], [35]. In tandem with this, Ogdh is also a major mitochondrial redox sensor modulating its activity in response to changes in the local redox environment. Redox signals like S-glutathionylation have been shown to control mitochondrial ROS production [8]. However, whether or not redox signals like S-glutathionylation control ROS production by Ogdh has remained unexplored. In this investigation, we found that S-glutathionylation reactions modulate ROS formation by Ogdh in a highly unexpected way. It was observed that GSSG and GSH control $O_{2}^{∙-}$/H_2_O_2_ production by Ogdh. The former lowered $O_{2}^{∙-}$/H_2_O_2_ formation by the enzyme complex while the latter had the opposite effect, amplifying $O_{2}^{∙-}$/H_2_O_2_ production at the expense of NADH formation. Grx2 reversed the GSH-mediated effects by deglutathionylating Ogdh. These findings indicate that Ogdh may play a crucial role in sensing overall changes in mitochondrial redox buffering networks which feedback to either amplify or reduce the production of ROS by this Krebs cycle enzyme. These findings are also consistent with observations that Pdh also senses changes in mitochondrial redox buffering networks which ultimately modulates its activity and capacity to produce ROS [23], [25].

It was confirmed that Ogdh can undergo S-glutathionylation when exposed to ≥1 mM GSSG which results in PSSG formation on the E_2_ subunit. GSSG-mediated S-glutathionylation resulted in a small but significant decrease in $O_{2}^{∙-}$/H_2_O_2_ formation during forward or reverse electron transfer. Incubation in 10 mM GSSG led to a further decrease in ROS formation which was associated with the increased S-glutathionylation of the E_2_ subunit. Recent work has found that Ogdh produces ROS from both the E_1_ and E_3_ subunits which accounts for the GSSG-mediated decrease in $O_{2}^{∙-}$/H_2_O_2_ formation when Ogdh is oxidizing either 2-oxoglutarate or NADH [22]. It is important to point out though that GSSG at 1 mM, which occurs at this concentration only during oxidative stress and when ROS formation is very high, only weakly inhibited Ogdh function and ROS production. In three separate studies, Szweda and colleagues provided empirical evidence that pre-oxidation of lipoamide by H_2_O_2_ is required to drive the S-glutathionylation of the E_2_ subunit [19], [28], [36]. It is therefore likely that H_2_O_2_-mediated formation of sulfenic acid on the E_2_ subunit is required to allow GSSG to more efficiently react with Ogdh. Thus, we can conclude that although GSSG can induce mild suppression of Ogdh ROS production, it does not seem likely that GSSG reacts directly with Ogdh in physiological conditions. Rather formation of reactive sulfenic acid moieties enhance S-glutathionylation through disulfide exchange with GSSG. Moreover, S-glutathionylation via a direct disulfide exchange reaction between thiols on Ogdh and GSSG most likely only occurs when mitochondria are faced with oxidative stress.

The mitochondrial redox buffering network is composed of a number of different redox pairs [37]. Due to its high concentration and negative redox potential glutathione is considered to be a major redox buffering agent in most biological environments including mitochondria [38]. Shifts in GSH/GSSG have been shown to modulate various cellular signaling programs including cell division, growth, energy metabolism, and gene expression [37], [39]. Enzymes like Pdh, a homolog of Ogdh, sense local changes in redox environment which ultimately modulates its activity and the amount of ROS it produces [23], [25]. As indicated above, a lower GSH/GSSG is typically associated with S-glutathionylation of target proteins which lowers cellular ROS formation, in particular in mitochondria [8]. Further, S-glutathionylation in mitochondria is required to protect enzymes like Ogdh and Complex I from irreversible deactivation due to over-oxidation by ROS [19], [40]. In consideration of the profound effects variations in GSH/GSSG can have on enzyme function, the impact of different GSH/GSSG ratios on Ogdh activity and ROS production was investigated in detail. We found that a low GSH/GSSG (0.1–1) slightly decreased Ogdh activity and $O_{2}^{∙-}$/H_2_O_2_ production. This is consistent with our results showing that GSSG alone can induce a small but significant change in Ogdh function. Unexpectedly, adjusting GSH/GSSG to 10 and 100 abolished Ogdh activity amplifying ROS production. Moreover, these effects were directly related to the concentration of GSH and not associated with fluctuations in GSH/GSSG. Indeed, we found that GSH directly modulated Ogdh activity and ROS production which was concentration dependent. At least 2.125 mM GSH was required to induce a 50% increase in ROS production and 2.819 mM for inhibition of NADH production. It is important to point out that GSH is not maintained at a consistent concentration in mitochondria. GSH levels are in a constant state of flux, increasing and decreasing in response to fluctuations in nutrient oxidation, H_2_O_2_ formation, and availability of NADPH [24]. Thus, based on our results, it follows that Ogdh activity and ROS production will also be in a state of flux, increasing and decreasing in response to local changes in GSH availability.

It is now becoming apparent that cells employ a rich panoply of systems to control ROS production to benefit from its signaling properties while simultaneously avoiding its toxic properties [41]. This includes various signaling pathways that includes electrophile signaling, the unfolded and heat shock response, and redox activated autophagy [41]. Redox signals also feedback on sites of production to control ROS formation and it is becoming increasingly clear that these signals can either increase or decrease $O_{2}^{∙-}$/H_2_O_2_ production. Here, we found that GSH S-glutathionylates Ogdh directly, specifically in the E_1_ subunit of the enzyme complex. It has been reported in several studies that GSH can S-glutathionylate proteins directly. This has been documented to occur in the cytosol and mitochondria, respectively, and typically requires formation of thiyl radicals, either on the protein being targeted for modification or on GSH itself [14]. For example, protein cysteine thiyl radical formation on several cysteine residues on Ndusf1 subunit drives the GSH-mediated S-glutathionylation of Complex I, even when GSH/GSSG is high [42]. Protein cysteine thiyl formation occurs when $O_{2}^{∙-}$ is generated in proximity to Complex I which prompts GSH-mediated S-glutathionylation and the further production of ROS [43]. It is possible that GSH-induced S-glutathionylation of Ogdh on its E_1_ subunit may be driven by a similar mechanism [44]. In fact, Ogdh generates a number of intrinsic thiyl radicals which inevitably results in its inactivation [45]. In this investigation, GSH S-glutathionylated the E_1_ subunit of Ogdh confirming the presence of redox modifiable cysteine residues. In addition, two previous studies have shown that E_1_ can undergo S-glutathionylation [29], [46]. It is also intriguing that GSSG and GSH induced S-glutathionylation on two different subunits indicating that both molecules have different targets for modulation of $O_{2}^{∙-}$/H_2_O_2_ formation by Ogdh. This would suggest that Ogdh utilizes its E_1_ and E_2_ subunits to differentially sense fluctuations in GSH and GSSG, respectively. While GSSG may block electron transfer to sites of production GSH has the opposite effect on the E_1_ subunit, amplifying ROS formation when Ogdh is metabolizing 2-oxoglutarate. Based on this it can be surmised that Ogdh senses local changes in GSH levels which may be associated with autooxidation of cysteine residues on the E_1_ subunit via thiyl radical formation.

Protein S-glutathionylation reactions have also been shown to enhance the formation of ROS. For instance, S-glutathionylation of nitric oxide synthase (NOS) uncouples the enzyme amplifying $O_{2}^{∙-}$ from its reductase subunit [47]. Intriguingly S-glutathionylation of NOS is mediated by protein thiyl radical formation and its subsequent reaction with GSH [47]. Similarly, prolonged S-glutathionylation of Complex I, for example when Grx2 is disabled or following chemical induction of S-glutathionylation by diamide, results in the amplification of ROS formation [15], [48]. In this study, GSH induced a robust increase in $O_{2}^{∙-}$/H_2_O_2_ formation by purified Ogdh when 2-oxoglutarate was being oxidized in the presence of NAD^+^. This was associated with S-glutathionylation of the E_1_ subunit and a complete loss of NADH formation. Similar observations were made in permeabilized liver mitochondria except NADH production was not abolished but significantly decreased. Intriguingly, we observed that GSH only induced a small decrease in ROS production during reverse electron transfer from NADH. In addition, supplementing reaction mixtures containing 2-oxoglutarate and NAD^+^ with KMV induced a significant drop in $O_{2}^{∙-}$/H_2_O_2_ production. These results coupled with the observation that GSH S-glutathionylates the E_1_ subunit of Ogdh indicate that GSH is likely amplifying ROS formation from the E_1_ subunit of Ogdh rather than the E_3_ subunit. Indeed, the E_3_ subunit is often considered the chief site for Ogdh mediated ROS formation however; recent evidence has shown that the E_1_ subunit also generates ROS through production of thiamine radicals within the subunit [22], [49]. Taken together, GSH modifies Ogdh resulting in diverted electron flow towards amplified ROS production from the E_1_ subunit.

GSH was also able to control Ogdh in the matrix of permeabilized liver mitochondria. We found that GSH redirected electron flow from 2-oxoglutarate amplifying $O_{2}^{∙-}$/H_2_O_2_ genesis which also lowered NADH production. In conjunction with these findings adding Grx2 to permeabilized mitochondria treated with 5 mM GSH reversed these effects. This indicates Grx2 can deglutathionylate Ogdh in the mitochondrial matrix. It is important to note that Grx2 lowered ROS production and restored NADH formation back to control levels which may be associated with the presence of endogenous NADPH-forming enzymes and GR. Only two Grx2 targets have been identified; Complex I and UCP3 [15], [17]. Grx2 is subjected to heavy regulation due to formation of inactive dimers via coordination of a 2Fe–2S cluster [33]. ROS-mediated disassembly of the Fe–S cluster results in the release of two Grx2 monomers harboring deglutathionylase activity [33]. Thus, GSH mediated diversion of electron flow in Ogdh and the amplification of ROS production would result in the activation of endogenous Grx2 creating a feedback loop resulting in the deglutathionylation of Ogdh and restoration of NADH production. Disabling this feedback loop may also have pathological consequences. Grx2-/- mitochondria generate higher than normal amounts of ROS which may be related to prolonged ROS production by Ogdh [15]. Knockout of Grx2 is associated with development of cardiac disease, neurological disorders, and cataracts [15], [50]. Grx2−/− also curtails cardiac, neural, and vascular development which is likely associated with disrupted cellular redox buffering and nutrient metabolism [51], [52], [53]. In addition, over production of ROS by either the E_1_ or E_3_ subunits of Ogdh has been implicated in neurological and cardiac disorders [18], [22]. Sensing mitochondrial redox buffering capacity has also recently been shown to modulate pyruvate dehydrogenase (Pdh) [23], [25]. Disabling mitochondrial redox circuits, chiefly through GSH depletion or disruption of NADPH formation, directly amplifies ROS formation by Pdh [23], [25]. Together, it would appear that both Ogdh and Pdh fulfill important redox sensing functions in mitochondria modulating ROS emission and carbon metabolism in response to fluctuations in redox buffering capacity. In light of the recently proposed Redox Code it is appropriate that both Ogdh and Pdh serve as sensors for mitochondrial redox buffering capacity since both enzymes serve as important entry points for carbon into the Krebs cycle and would thus influence overall redox signals throughout mitochondria and in the cell [24].

The present findings reveal a novel signaling mechanism which integrates mitochondrial redox buffering into control over mitochondrial ROS production in a unique and highly unexpected way. This network is centralized around the Ogdh-mediated sensing of GSH levels which, if high enough, can divert electron flow away from NADH production and oxidative metabolism towards ROS formation. To our knowledge this is the first demonstration that a reduced antioxidant like GSH can work in the opposite direction in mitochondria-amplifying ROS formation instead of simply quenching it. Our findings also illustrate the fundamental complexities surrounding redox signaling and how ROS production, antioxidant defense, redox buffering, and oxidative metabolism are heavily integrated into one another [24]. We also found that Grx2 is able to reverse these effects, deglutathionylating Ogdh which lowers ROS formation and restores electron flux back to normal. It is tempting to speculate that glutathione sensing may have been tempered to ensure that ROS production can be amplified in times when antioxidant capacity is high. This would ensure the continued use of ROS as a signaling molecule while simultaneously controlling its production, either through antioxidant systems or via feedback loops that are activated and deactivated by changes in redox environment.

## Conflict of interest

The authors declare that they have no conflicts of interest with the contents of this article.

## Author contributions

RJM conceived the study, designed the experiments, and wrote the article. Performed and analyzed the results for Fig. 2, Fig. 3, Fig. 4, Fig. 5, Fig. 6, Fig. 7 and designed Fig. 1 and the Graphical abstract. SLC and DCA provided technical assistance and edited the manuscript. All authors reviewed the results and approved the final version of the manuscript.
